# Generation and characterization of rat liver stem cell lines and their engraftment in a rat model of liver failure

**DOI:** 10.1038/srep22154

**Published:** 2016-02-26

**Authors:** Ewart W. Kuijk, Shauna Rasmussen, Francis Blokzijl, Meritxell Huch, Helmuth Gehart, Pim Toonen, Harry Begthel, Hans Clevers, Aron M. Geurts, Edwin Cuppen

**Affiliations:** 1 Hubrecht Institute, KNAW and University Medical Center Utrecht, 3584CT Utrecht, The Netherlands; 2 University Medical Center Utrecht, PO Box 85060, 3508 AB Utrecht, The Netherlands; 3 Department of Physiology, Medical College of Wisconsin, 8701 Watertown Plank Road, Milwaukee, WI 53226, USA; 4 Wellcome Trust/MRC Stem Cell Institute, Department of Physiology, Development and Neuroscience, University of Cambridge, Tennis Court Rd, Cambridge CB2 1QN, UK; 5 Wellcome Trust/Cancer Research UK Gurdon Institute, University of Cambridge, Tennis Court Rd, Cambridge CB2 1QN, UK; 6 Human and Molecular Genetics Center, Medical College of Wisconsin, 8701 Watertown Plank Road, Milwaukee, 53226, WI, USA

## Abstract

The rat is an important model for liver regeneration. However, there is no *in vitro* culture system that can capture the massive proliferation that can be observed after partial hepatectomy in rats. We here describe the generation of rat liver stem cell lines. Rat liver stem cells, which grow as cystic organoids, were characterized by high expression of the stem cell marker *Lgr5*, by the expression of liver progenitor and duct markers, and by low expression of hepatocyte markers, oval cell markers, and stellate cell markers. Prolonged cultures of rat liver organoids depended on high levels of WNT-signalling and the inhibition of BMP-signaling. Upon transplantation of clonal lines to a *Fah*^−/−^ *Il2rg*^−/−^ rat model of liver failure, the rat liver stem cells engrafted into the host liver where they differentiated into areas with FAH and Albumin positive hepatocytes. Rat liver stem cell lines hold potential as consistent reliable cell sources for pharmacological, toxicological or metabolic studies. In addition, rat liver stem cell lines may contribute to the development of regenerative medicine in liver disease. To our knowledge, the here described liver stem cell lines represent the first organoid culture system in the rat.

The adult liver has a vital role in metabolism, detoxification and the production of essential serum proteins. In contrast with the high regenerative capacity of the rat liver^1^,^2^,^3^,^4^, isolated rat liver cells have limited potential to proliferate *in vitro*^5^. The highest growth potential of rat liver cells in culture has been attributed to a hepatocyte subpopulation called small hepatocytes. Expansion in this culture system is limited to a period of 1–2 weeks after isolation^6^.

As a consequence, toxicological and pharmacological studies still depend on primary cultures of rat hepatocytes^7^,^8^,^9^. The high functional variability and limited life span of primary hepatocytes pose limitations to these studies^7^. The derivation of rat liver stem cell (LSC) lines could help circumvent these issues and contribute to the minimization of experimental animal usage^10^.

We previously described culture conditions that sustain the long-term culture of adult mouse stem cells from the intestine^11^, stomach^12^, liver^13^ and pancreas^14^. In addition, we developed culture systems that support the self-renewal of stem cells from the adult human intestine^15^,^16^ and the human liver^17^. These cultures grow as 3-dimensional organ buds commonly referred to as organoids. Self-renewal of these organoid cultures depends on the Lgr5-ligand and WNT agonist RSPO1. In self-renewing epithelial organ systems such as the small intestine, colon, and the stomach, Lgr5 is expressed in the stem cells that reside at the bottom of the crypts^18^. In contrast with the gastrointestinal tract, other organ systems such as the pancreas and the liver lack actively dividing stem cells. The stem cells in these organs become activated after tissue damage to support regeneration. Several recent studies using different animal models of liver damage demonstrate that multiple cell types can contribute to liver regeneration, such as hepatocytes^19^,^20^,^21^,^22^, duct-derived oval cells^23^,^24^,^25^ and hepatic stellate cells (a liver-resident mesenchymal cell)^26^. Apparently, different cell types of the adult liver can act as facultative stem cells and contribute to liver regeneration, depending on the type and extent of the damage^23^,^27^. Liver and pancreas regeneration involves activation of the WNT-pathway^13^,^14^. Genetic lineage tracing techniques have recently demonstrated that Lgr5, which is not expressed in the healthy adult mouse liver or pancreas, becomes activated in a subpopulation of cells upon injury^13^,^14^. In the damage mouse liver, small Lgr5 + cells appear and contribute to the generation of hepatocytes and ductal cells, indicating that the Lgr5^+^cells have bipotential.

In the presence of the Lgr5-ligand RSPO1, single Lgr5^+^ mouse liver cells can give rise to cystic organoid cultures that can be expanded indefinitely, while maintaining the potential to differentiate towards hepatocytes and duct cells, demonstrating that liver derived Lgr5^+^ cells are true bi-potential stem cells *in vitro*. Moreover, *in vitro* differentiated liver stem cells are able to engraft in an Fah^−/−^ mouse model of liver failure, where they develop into functional hepatocytes^13^. More recently, we also described culture conditions that support human liver stem cell cultures^17^.

The liver organoid system is considered a valuable tool for drug testing^10^. Considering the established role of the rat in pharmacological research we pursued the development of an organoid culture system for the rat liver in the current study. We chose for the SHR/OlaIpcv (SHR) and BN-Lx/Cub (BN-Lx) rat strains, because these are the two founder strains of the rat HXB/BXH recombinant inbred panel that has been extensively phenotyped at the physiological, behavioral and molecular levels and all genomic variation between both strains has been identified^28^,^29^.

## Results

### Establishment of rat liver stem cell lines

**Figure 1:**
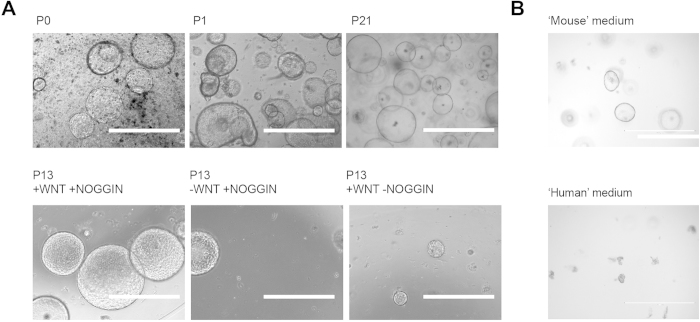
Establishment of rat liver stem cells and the effects of various growth factor conditions on the cultures. (**A**) Rat liver stem cells grow as cystic organoid structures (top panels), which are lost when NOGGIN or WNT3A is absent from the medium (bottom panels). Regular cultures contained 50% conditioned medium (produced in house) of WNT3A and 10% conditioned medium (produced in house) of Noggin. For conditions lacking WNT3A or NOGGIN we used medium conditioned on control cell lines that do not produce these factors. (**B**) Human liver stem cell medium fails to support the self renewal of rat liver stem cells. Scale bars are 1mm.

For the generation of mouse and human liver stem cell lines, isolated duct cells are initially subjected to high levels of WNT-signaling and inhibition of BMP-signaling by Noggin during the first 3–4 days of culture^13^,^17^. After culture induction, WNT and Noggin are no longer required. To establish rat liver stem cell lines, liver tissue was digested with collagenase and differential centrifugation steps were performed to enrich for duct cells. The fractions containing rat duct cells were embedded in matrigel and cultured in mouse liver stem cell culture initiation conditions^13^, which includes 50% conditioned medium (produced in house) of WNT3A and 10% conditioned medium (produced in house) of Noggin. After 2 days the first cystic epithelial organoids appeared reminiscent of mouse and human liver stem cells (Fig. 1A). In contrast with the mouse, human liver stem cells are routinely cultured in the presence of 2 small chemical compounds: forskolin (a cAMP pathway agonist) and A83-01 (an inhibitor of the Tgf-ß receptors Alk4/5/7). However, when rat liver cells were subjected to these ‘human’ liver stem cell conditions^17^, cystic organoids were lost within 1 week after switching culture conditions, indicating that these conditions fail to support rat liver stem cell self-renewal (Fig. 1B).

### Rat liver stem cell self-renewal depends on WNT and NOGGIN

**Figure 2:**
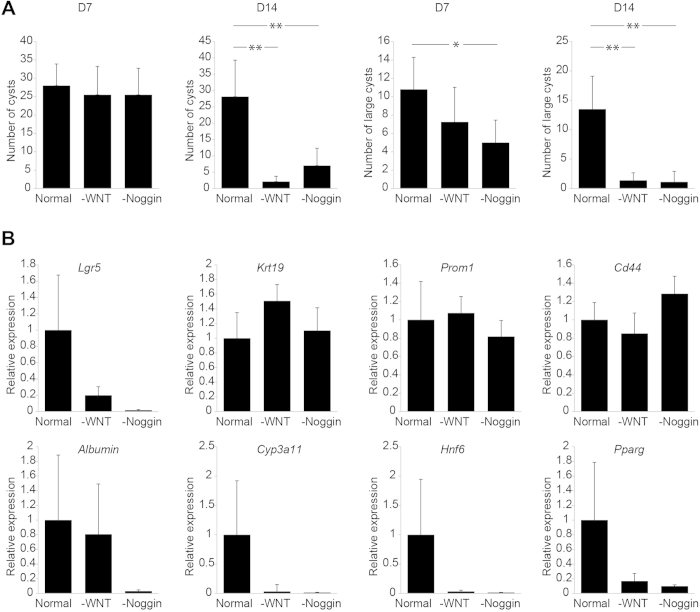
Effects of growth factor conditions on the number of organoids and on gene expression levels. (**A**) Quantification of the number of cystic organoids (top panels) or the number of large cystic organoids upon 7 and 14 days of culture in the absence of WNT or NOGGIN. Results are expressed as mean ± SEM of three independent cultures. (**B**) The effects of the absence of WNT or NOGGIN on the expression levels of stem cell/duct markers and hepatocyte markers. Results are expressed as mean ± SEM. Asterisks denote significant differences (2-tailed student’s t-test): *p < 0.05; **p < 0.01.

In the presence of WNT and NOGGIN, the cysts continued to grow and they were split 10–12 days after culture initiation. Subsequent passages were performed at 6–9 day intervals at 1:4–1:8 split ratios. The cultures could be maintained beyond passage 25 without signs of senescence or loss of self-renewal potential. Withdrawal of Noggin or WNT had adverse effects on the cultures, drastically reducing the number of cysts after 14 days of culture (Fig. 2A). These effects were already noticeable at day 7 of Noggin or WNT withdrawal, reducing the number of large cysts at this time point (Fig. 2B). Therefore, for rat, but not mouse, WNT and NOGGIN are essential to sustain self-renewal *in vitro*. Quantitative RT-PCR revealed that in the absence of WNT or NOGGIN, the stem cell marker *Lgr5*^13^ was downregulated, while the expression levels of the duct marker *Krt19*^30^ and the bipotent hepatic progenitor markers *Cd44*^31^ and *Prom1*^32^ remained high (Fig. 2B). This coincided with a downregulation of the hepatocyte markers *Albumin* and *Cyp3a11* and also of the hepatocyte maturation markers *Hnf6* (*OC-1*) and *Pparg*. These data suggest that in the absence of WNT or NOGGIN, stem cells are lost from the cultures through differentiation towards duct cells, but not towards hepatocytes.

### Rat liver stem cell lines express stem cell markers and duct markers

**Figure 3:**
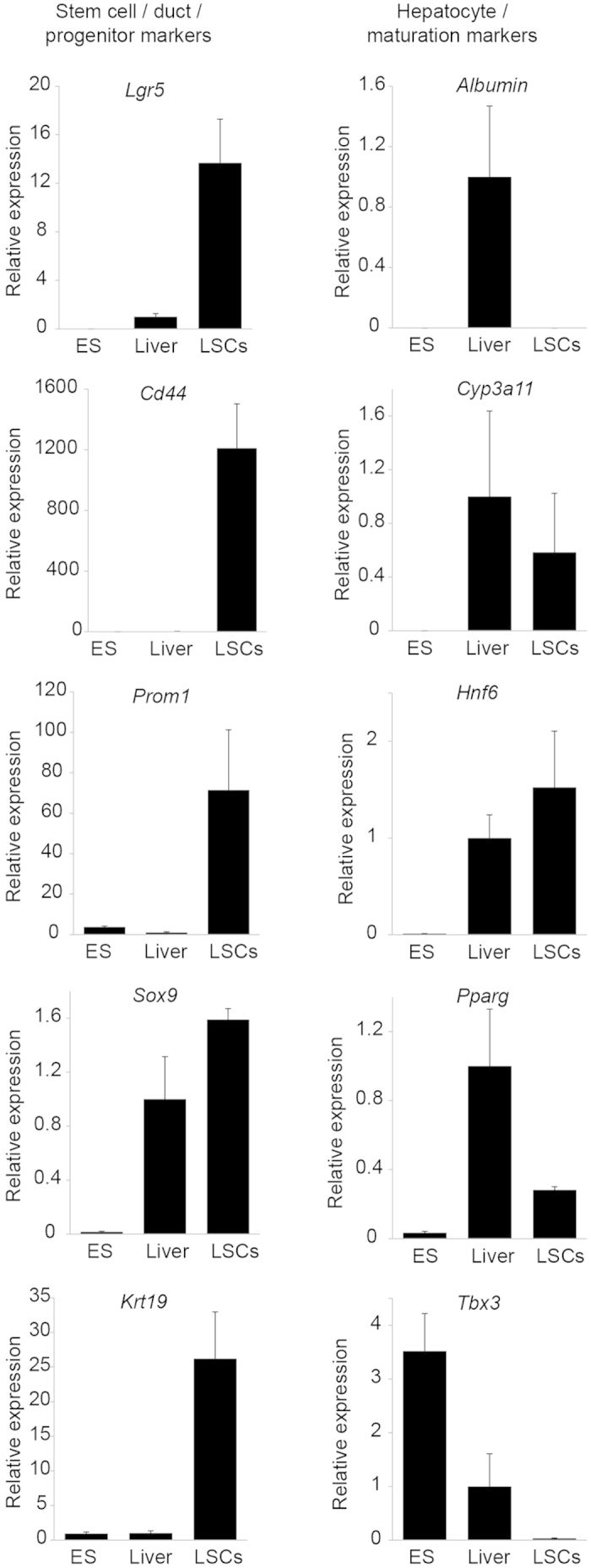
Characterization of rat liver stem cell cultures by quantitative RT-PCR. Relative expression levels of stem cell/duct markers in embryonic stem cells, liver, and liver stem cells (left panels). Expression levels of hepatocyte/maturation markers in embryonic stem cells, liver, and liver stem cells (right panels). Results are expressed as mean ± SEM. ES = rat embryonic stem cells.

The rat liver stem cells were further characterized by the expression levels of markers for various liver cell types (Fig. 3). In clonally expanded cell lines, the expression level of the stem cell marker *Lgr5* was higher compared to the expression in the liver or in the rat embryonic stem cell line DA27. The expression of the liver progenitor markers *Cd44*, *Prom1*, and *Sox9*^33^ was enriched in the rat liver stem cell lines. *Krt19* was also expressed at relatively high levels, probably reflecting the ductal origin of the liver stem cell lines. The hepatocyte maturation markers *Pparg* and *Tbx3*^34^ and the hepatocyte markers *Albumin* and *Cyp3a11* were expressed at lower levels in the rat liver stem cell cultures when compared to the expression levels in the liver (Fig. 3).

### RNA-seq characterization of rat liver stem cell lines

**Figure 4:**
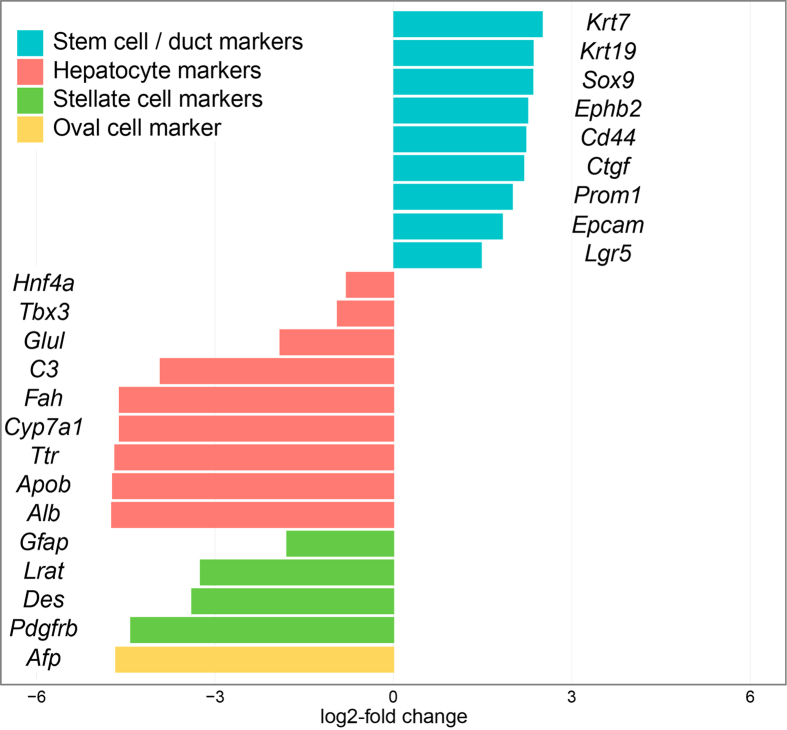
Differential expression between liver samples and liver stem cell samples of selected markers of liver cell types. Log2 fold changes of stem cell /duct markers, hepatocyte markers, stellate cell markers, and the oval cell marker *Afp*. Negative fold changes indicate lower expression in rat liver stem cells compared to the liver, positive fold changes indicate higher expression in rat liver stem cells compared to the liver. All markers are significantly differently expressed (adjusted *P*-value < 0.05).

We performed RNA-seq on 7 stem cell clones and 4 liver samples to further characterize the rat liver stem cell cultures. Analysis of the RNA-seq data confirmed the significantly higher expression in rat liver stem cells of the stem cell marker *Lgr5* and several duct/progenitor including *Cd44*, *Prom1*, *Krt19* and *Sox9* (Fig. 4). Hepatocyte markers such as *Albumin*, *Fah*, and *Hnf4a* were expressed at significantly lower levels in the liver stem cells compared to the liver. The same was true for a selection of markers for stellate cells and oval cells (Fig. 4).

Gene functional classification and clustering analyses with DAVID^35^,^36^ of all 396 significantly higher expressed genes revealed that rat liver stem cells are enriched for keratin family genes, genes involved in plasma membrane function and genes involved in calcium binding (see Supplemental file S3). KEGG pathway analysis further revealed significantly higher expression of genes involved in extra-cellular matrix-receptor interactions (Fold Enrichment = 6.5, adjusted *P*-value = 0.018, FDR = 0.2), such as several integrins (*Integrin α2*, *Integrin α3*, *Integrin β4*, and *Integrin β6*) and laminins (*Laminin α5*, *Laminin β3*, and *Laminin γ2*) (see Supplemental file S3). Subsequently, gene functional classification and clustering analyses were performed on all 2276 genes that were expressed at significantly lower levels in the rat liver organoid cultures. These analyses revealed that many low-expressed genes play roles in mature liver function, such as genes involved in complement activation, protein synthesis, and family members that belong to the Cytochrome p450 family (see Supplemental file S4). KEGG analysis also showed a significant low expression of genes involved in liver function such as genes involved in complement and coagulation cascades (Fold enrichment = 4.8, adjusted *P*-value = 2.0E-19, FDR = 1.4E-18), drug metabolism (Fold enrichment = 4.1, adjusted *P*-value = 2.7E-13, FDR = 3.8E-12), PPAR signaling (Fold enrichment = 3.9, adjusted *P*-value = 5.2E-12, FDR = 1.1E-10), and fatty acid metabolism (Fold enrichment = 4.4, adjusted *P*-value = 4.8E-09, FDR = 1.3E-07) (see Supplemental file S4).

### *In vitro* differentiation of rat liver stem cell lines

**Figure 5:**
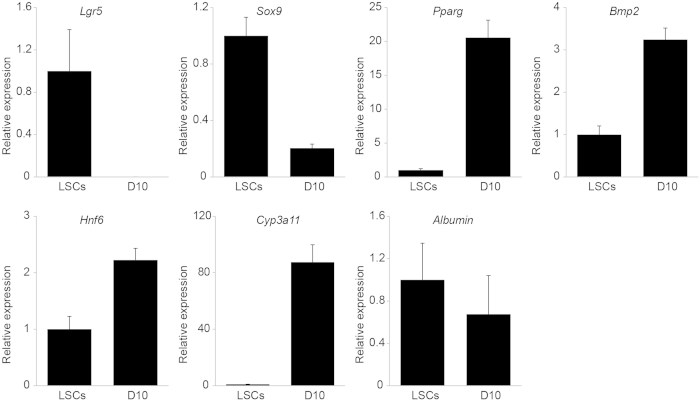
Effect of *in vitro* differentiation conditions on rat liver stem cell cultures as determined by quantitative RT-PCR. Relative expression levels of stem cell/duct markers and hepatocyte/maturation markers in undifferentiated liver stem cells (LSCs) and at day 10 (D10) of differentiation. Results are expressed as mean ± SEM.

Mouse liver stem cells can be differentiated through the inhibition of Notch and TGF-ß, which results in enhanced hepatocyte cell fate^13^. When these *in vitro* differentiation conditions were applied to rat liver stem cells, the stem cell/progenitor markers *Lgr5* and *Sox9* were downregulated, while expression of the liver maturation markers *Pparg*, *Bmp2*, and *Hnf6* was upregulated (Fig. 5). Furthermore, the expression of the mature hepatocyte marker *Cyp3a11* was increased upon differentiation. *Albumin* expression was not upregulated, indicating that under these *in vitro* differentiation conditions, the rat liver stem cells have embarked on a path towards hepatocyte cell differentiation, but they have not fully matured in *Albumin*-expressing hepatocytes.

### *In vivo* differentiation of rat liver stem cells

**Figure 6:**
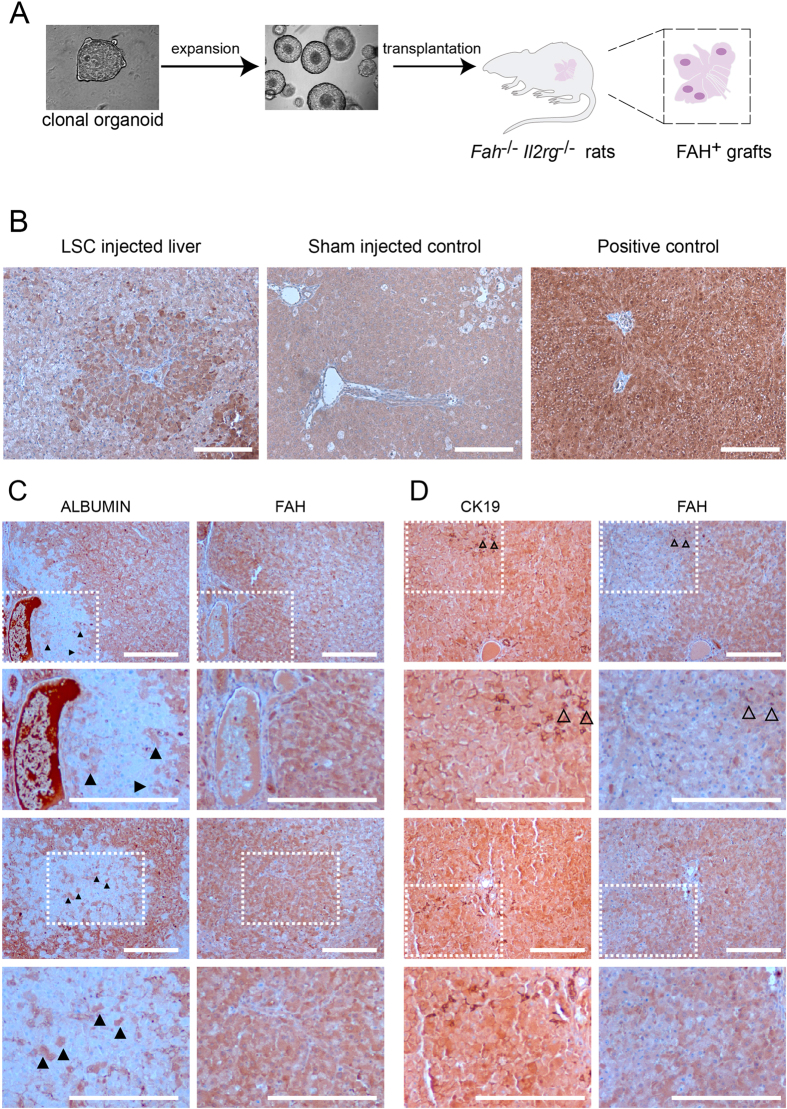
Transplantation of rat liver stem cells to the *Fah*^−/−^ *Il2rg*^−/−^ rat model of liver failure. (**A**) Experimental set-up: single organoids were manually picked and expanded until enough cells were obtained for intrasplenic transplantation into the *Fah*^−/−^ *Il2rg*^−/−^model of liver failure. After transplantation the rats were cycled on and off NTBC (see material and methods section for more detail). Within 2 months after transplantation rat livers were analyzed for engraftment by immunohistochemistry. (**B**) Immunohistochemical analysis of FAH-expression demonstrates engraftment in livers of animals that received liver stem cells. (**C**) Consecutive sections of FAH-positive areas were analyzed for the expression of the hepatocyte marker Albumin. The expression of FAH and Albumin is nearly mutually exclusive, indicating that the majority of the injected cells have not yet fully differentiated to hepatocytes. Caged boxes with dashed lines are enlarged in the panels below. Arrowheads indicate a subset of ALBUMIN-positive hepatocytes in the FAH-positive grafts. (**D**) Consecutive sections of FAH-positive areas were analyzed for the expression of the duct marker CK19. In addition to the ducts, CK19 positive cells were also detected inside the FAH-positive grafts outside the ducts (open arrowheads). Scale bars are 200 μm.

Differentiation towards hepatocytes may be more efficient when the cells are subjected to the cues such as present in the damaged liver. Therefore, we tested the potential of rat liver stem cells to engraft into the livers of fumarylacetoacetate hydrolase (*Fah*) interleukin 2 receptor gamma (*Il2rg*) double mutant rats, a model for tyrosinemia type I liver disease. *Fah*^−/−^ hepatocytes accumulate intermediate metabolites resulting in programmed cell death and ultimately in liver failure unless the mutant rats are administered NTBC (2-(2-nitro-4-trifluoro-methylbenzyol)-1,3-cyclohexanedione). Clonally expanded organoids from the BNLx and the SHR strains were expanded further to obtain enough cells for transplantations. Single cell suspensions were injected intrasplenically in the rats (Fig. 6A). Sham injected animals served as controls. All animals were cycled on and off NTBC to induce liver damage and to facilitate engraftment and expansion of transplanted cells. Subsequently, engraftment was analyzed by immunohistochemical staining of liver sections with an antibody against FAH (Fig. 6B). FAH positive cells were detected in 3 out of 7 transplanted rats. In one liver, the engrafted cells were of BNLx origin and the sizes of the grafts were calculated to comprise ~20% of the liver. In the remaining 2 livers, the engrafted cells were of SHR-origin and the engrafted areas were calculated to be 1.5% in one liver and less than 1% in the other. To determine the differentiation potential of the engrafted cells consecutive sections were stained with an antibody that recognizes rat Albumin. Albumin staining was readily detected outside the FAH-positive patches resulting in nearly mutually exclusive expression patterns between Albumin and FAH (Fig. 6C). Importantly, there were Albumin positive cells present in the FAH-positive areas, indicating the potential of engrafted rat liver stem cells to differentiate towards hepatocytes. Finally, we also observed CK19 positive cells outside the ducts in the FAH-positive engrafted areas (Fig. 6D). The CK19 positive cells possibly highlight engrafted cells that are migrating towards damaged tissue.

## Discussion

In the current study, we demonstrate that self-renewing organoids can be generated from the rat liver. To our knowledge, these represent the first organoid culture system in the rat. Unlike mouse and human liver organoid cultures, rat liver stem cells require high levels of WNT-signaling and suppressed BMP-signaling. Mouse and human liver stem cells, on the other hand, only require WNT and NOGGIN during culture initiation and not thereafter. This discrepancy may reflect species differences in liver regeneration^37^.

Upon transplantation, rat liver stem cells differentiated towards FAH positive grafts containing CK19 and Albumin positive cells, indicating that rat liver stem cells have the bipotential to differentiate into either hepatocytes or duct cells. Albumin expression was observed in the minority of the engrafted cells indicating incomplete differentiation of the transplanted stem cells at the examined time points.

Mouse and human liver stem cells can be differentiated towards albumin positive hepatocytes. Under mouse hepatocyte differentiation conditions, rat liver stem cells did not adopt a hepatocyte phenotype. The high levels of WNT and NOGGIN required for rat liver stem cells may adversely affect their differentiation potential towards hepatocytes. Nevertheless, the transplantation experiments demonstrate the potential of rat liver stem cells towards hepatocytes. The *in vitro* differentiation conditions of rat liver stem cells towards hepatocytes needs further optimization.

The stem-cell model of liver regeneration has recently been challenged. Several studies using different mouse models of liver damage demonstrate that liver regeneration doesn’t depend on the activation of liver-specific stem cells, but on the proliferation of hepatocytes^19^,^20^,^21^,^22^. On the other hand, studies in the rat have demonstrated that when hepatocyte proliferation is blocked, partial hepatectomy leads to the appearance of duct-derived oval cells that gradually give rise to small hepatocytes and ultimately hepatocytes, resulting in the restoration of liver function^23^,^24^,^25^. In addition, hepatic stellate cells (a liver-resident mesenchymal cell) can also contribute to liver regeneration^26^. Apparently, different cell types of the adult liver can act as facultative stem cells and contribute to liver regeneration, depending on the type and extent of the damage^23^,^27^. As their mouse and human counterparts^13^,^17^, the here described duct-derived rat liver organoids can be cultured extensively (>25 passages) and differentiate into duct cells and hepatocytes, thereby fulfilling the two main criteria of a bona fide stem cell: i.e. self-renewal capacity and differentiation potential^38^,^39^.

Mouse and human liver stem cells are derived from biliary epithelial duct cells^13^,^17^. To establish the rat liver stem cell lines described in the current study, bulk liver cell populations were enriched for duct cells. The ductal origin of the rat liver stem cell cultures was further reflected by the gene expression patterns obtained from quantitative RT-PCR and RNA-seq experiments. Rat liver stem cell cultures expressed duct and stem cell markers such as *Krt19*, *Sox9*, *Cd44*, *Epcam*, and *Lgr5*. The relative low expression of markers for hepatocytes, stellate cells, and oval cells, decrease the likelihood that the rat liver stem cell cultures originated from these liver cell types. RNA-seq analysis also revealed enhanced expression in the rat liver stem cells of genes that belong to the families of integrins and laminins, which are involved in extracellular matrix-receptor interactions. The integrin and laminin genes that are highly expressed in rat liver stem cells are also known to be expressed in mouse and human biliary epithelial duct cells^40^,^41^. This finding emphasizes the ductal phenotype of the rat liver stem cell cultures and further supports their ductal origin.

Interestingly, genes encoding the calcium binding proteins, including S100 calcium-binding proteins A4 and A6, were enriched in rat liver stem cells. S100A4 and S100A6 are thought to play important roles in the activation of stem cells at the onset of hair follicle regeneration^42^. Their elevated expression in liver stem cells may indicate a role for these factors in rat liver stem cells.

Here, we have demonstrated that rat duct cells can self-renew extensively in culture, while having the capacity to engraft into the liver of a rat model of liver failure, where they can differentiate towards hepatocytes. Rat liver stem cell lines may contribute to the development of regenerative medicine in liver disease. Furthermore, rat liver stem cell lines may be particularly useful for studies on rat models for human metabolic disorders, such as the Gunn rat (model for CN syndrome typeI), the FAH^–/–^ (model of tyrosinaemia type I), the rat model for hemochromatosis, and the Long Evans Cinnamon rat (model of Wilson’s disease)^43^,^44^,^45^. These models could benefit from rat liver stem cell lines as a tool to further elucidate the molecular mechanisms of these diseases or to examine the potential of liver stem cells in restoring liver function upon transplantation. Moreover, rat liver stem cells hold potential as an additive or alternative model to primary rat hepatocytes, which are currently frequently used in pharmacological studies. We think these rat liver stem cell lines will be particularly valuable, because they were generated from the well-characterized founder strains of the rat HXB/BXH recombinant inbred panel^28^,^29^.

## Methods

### Animal experiments

All animals received human care and all animal procedures and breeding were performed in accordance with the institutional review committees at the Hubrecht Institute and the Medical College of Wisconsin. These animal studies were performed under protocols approved either by the Institutional Animal Care and Use Committee of MCW or by the animal ethical committee (DEC) of the Netherlands Academy of Sciences (KNAW), the Netherlands (Protocol nr: H1 11 08.02). Animals with the Fah mutation and IL2RG deficiency were maintained on drinking water containing 16 mg/L (2-(2-nitro-4-trifluoromethylbenzoyl)-1,3-cyclohexanedione) (NTBC) (a kind gift from Yecuris, Inc.) and Sulfatrim at a dose of 56 mg/L.

### Rat liver stem cell isolation and culture

All chemicals were obtained from Sigma Aldrich (Zwijndrecht, the Netherlands) unless stated otherwise. A lobe of freshly isolated liver was cut into small pieces that were resuspended in collagenase solution consisting of 0.02% Collagenase type IV in freshly prepared Krebs solution (120 mM NaCl, 4.8 mM KCl, 1.2 mM KH_2_PO_4_, 1.2 mM MgSO_4_.7 H_2_O, 24 mM NaHCO_3,_ 1.3 mM CaCl_2,_ and 20 mM HEPES). After a 10 minute incubation at 37 °C the cell suspension was passed through a 40 μm cell strainer and diluted 4-fold with ice-cold Krebs solution. The cell suspension was centrifuged at 60 g and the supernatant was centrifuged at 300 g. The resulting cell pellet was washed once in Advanced DMEM/F12 (Life Technologies, Bleiswijk, the Netherlands) followed by another 5 min centrifugation at 300 g. The cell pellet was resuspended in ice-cold matrigel and plated as 50 μl droplets of a warm 24 well plate. After the matrigel had solidified the culture medium was added to the wells. Culture medium consisted of Advanced DMEM/F12, 1.25 mM n-acetylcysteine, 50% WNT3a conditioned medium (produced in house), 10% RSPO1 conditioned medium (produced in house), 10% NOGGIN conditioned medium (produced in house), 50 ng/ml EGF (Peprotech, London, United Kingdom), 10 nM gastrin (Sigma-Aldrich), 100 ng/ml Fgf10 (Peprotech), 10 mM nicotinamide (Sigma-Aldrich), 50 ng/ml HGF (Peprotech), and 10 μM Y-27632 (ENZO Life Sciences, Raamsdonksveer, the Netherlands). Culture medium also contained HEPES (Life Technologies), B27 supplement without retinoic acid (Life Technologies), and N2 supplement (Life Technologies). The same medium was used to test the effects of WNT or NOGGIN depletion, except for that WNT or NOGGIN conditioned media were replaced with media conditioned on control cell lines that do not produce these factors. Rat liver stem cells were cultured in a humidified tissue culture incubator with 5%CO_2_ and atmospheric O_2_ at 37 °C. Medium was refreshed every other day and the cells were mechanically split every 6–9 days at 1:4–1:8 split ratios. Clonal lines were obtained after manually picking and expanding single organoids. For *in vitro* differentiation, rat liver organoids were seeded and kept 2–4 days under regular medium. Then the medium was replaced with medium containing A8301 (50 nM, Tocris Bioscience) and DAPT (10 nM,Sigma-Aldrich) and lacking RSPO1, HGF and nicotinamide. Medium was changed every other day for a period of 10 days.

### Transplantations

Fah^−/−^; Il2rg^−/−^ animals 4–5 weeks of age were placed on 2 mg/L NTBC two days prior to transplantation. At the day of transplantation the animals were anesthetized by isoflurane inhalation. Single cell suspensions containing ~2.5 × 10e6 liver stem cells in 500 μl of 0.9% saline were injected into the tip of the spleen using a 27 gauge needle. Controls animals received saline alone. Animals were kept on 2 mg/L NTBC for one week following transplantation to allow for engraftment and maturation of cells. Animals were then removed from NTBC for 2 days, followed by 4 days of 16 mg/L NTBC administration. The animals were then cycled off/on NTBC according to 15% weight loss. 15% weight loss was calculated from the day of withdrawal weight or the day of highest achieved body weight. Animals were weighed and distress scored daily until they reach 15% weight loss or a distress score of six upon which they were returned to NTBC-Sulfatrim supplemented water for five days at a time. After five days, animals were removed from NTBC-Sulfatrim supplemented water and 15% weight loss was calculated again. This cycle was repeated as needed for each individual animal until the end of the experiment.

### Immunohistochemistry

Formalin-fixed and paraffin embedded liver tissues were deparaffinized followed by blocking of endogenous peroxidase activity. Antigen Retrieval was performed by boiling the slides for 30 minutes in citrate buffer (pH 6.0). Non-specific binding sides were blocked with normal goat serum prior to incubation with the primary antibody solution. The following primary antibodies (all from Bio-Connect, Huissen, the Netherlands) were used: rabbit anti-FAH (cat. no. ARP41681_T100) rabbit anti-Cytokeratin 19 (cat. no. 14965-1-AP) and rabbit anti-Albumin (cat. no. 16475-1-AP). After the wash steps following the primary antibody step, the sections were incubated in HRP conjugated anti-rabbit (DAKO envision system K4003, Heverlee, Belgium). Antibody binding was visualized by incubating the sections with DAB substrate and the sections were counterstained with hematoxylin before the slides were mounted. To quantify the engraftment areas, 10 random areas were designated per slide and in these areas graft sizes were measured based on FAH expression. Since some of the transplanted cells probably are FAH negative, these values likely represent underestimates of the true engraftment.

### Quantitative RT-PCR

RNA extraction from rat liver stem cells was performed with Trizol (Life Technologies) according to the manufacturer’s protocol. RNA was reverse transcribed with M-MLV reverse transcriptase (Promega, Leiden, the Netherlands) using random primers. The resulting cDNA was used as a template in real time PCR reactions. PCR reactions were prepared according the SYBR green supermix protocol (Bio-Rad, Veenendaal, the Netherlands) using intron-spanning primer pairs (Table 1). RT-PCR reactions were run on CFX 96 and CFX 384 real-time PCR detection systems (Bio-Rad). PCR amplification cycles were preceded by a 3 minute step at 95.0 °C followed by 40 cycles at 95.0 °C, 58.0 °C, 72.0 °C, and 80 °C (real-time measurement) respectively. Melt curves were generated after PCR amplification. The threshold cycles were used to calculate relative expression levels according to the ∆∆Ct method with GAPDH as a reference gene.

### RNA-sequencing

RNA extraction from 7 clones of rat liver stem cells (3xSHR, 4xBNLx) and 4 liver samples (2x SHR and 2xBNLx) was performed with Trizol (Life Technologies) according to the manufacturer’s protocol. The isolated RNA was subjected to poly (A) selection with the MicroPoly(A) Purist Kit (Life Technologies) and CAP-selection with the mRNA ONLY Eukaryotic mRNA isolation kit (Epicentre/Illumina, Eindhoven, the Netherlands). Next, the CAP and poly (A) selected RNA was heat sheared followed by hybridization and ligation to the SOLID adapters according to the SOLID sequencing protocol. The RNA was reverse transcribed using the SOLID RT primer followed by size-selection of the complementary DNA (cDNA). The cDNA was subsequently amplified using a SOLID PCR primer and a unique barcoding primer for each library. Sequencing was performed on a SOLID Wildfire. The dataset is publicly available at the European Nucleotide Archive (ENA): http://www.ebi.ac.uk/ena/data/view/PRJEB9910.

### RNA-seq data analysis

RNA-seq reads were mapped with Burrows-Wheeler Aligner 0.5.9^46^ onto the rat reference genome RGSC5.0. The BAM files were sorted with Sambamba v0.5.1^47^ and reads were counted with HTSeq-count version 0.6.0 (default settings)^48^ to exons as defined in Rattus_norvegicus.Rnor_5.0.74.gtf (Ensembl). Non-uniquely mapped reads were not counted, due to their alignment quality of 0. Subsequently, DESeq version 1.16.0^49^ was used to normalize counts. The non-zero normalized gene counts showed a bimodal distribution, and a gene read count threshold of 50 was used to select expressed genes (see Supplemental Fig. S1). Hierarchical clustering was performed using the Spearman’s rank correlation of the normalized counts of expressed genes and divides liver samples and liver stem cell samples into two distinct groups (see Supplemental Fig. S2). DESeq nbinomTest was used to test for differential expression between liver and LSC samples. We selected differentially expressed genes with an adjusted *P*-value < 0.05 (Benjamini-Hochberg FDR correction) and an absolute log2 fold-change>2. The resulting 2672 genes were divided into a set of 396 genes higher expressed in the rat liver stem cells compared to the liver and a set of 2276 genes expressed at lower levels in the LSCs. These gene sets were functionally annotated using DAVID^35^,^36^.

## Additional Information

**How to cite this article**: Kuijk, E. W. *et al.* Generation and characterization of rat liver stem cell lines and their engraftment in a rat model of liver failure. *Sci. Rep.* **6**, 22154; doi: 10.1038/srep22154 (2016).

## Supplementary Material

Supplementary Information

## Figures and Tables

**Table 1:** List of primers.

Gene	Forward Primer	Reverse Primer
*Gapdh*	TGACTCTACCCACGGCAAGTTCAA	ACGACATACTCAGCACCAGCATCA
*Hnf6*	CCGGAGGATGTGGAAGTGG	AGCTGCTGGGAAATGGTGA
*Krt19*	CTAATGGCGAGCTGGAGGTGAAG	GGCGGGCATTGTCGATCTGTAGGA
*Lgr5*	AGATGAGCGGGACCTTGAA	ATTACCCCGATTAGCAGTTTTATG
*Bmp2*	ATCCGGCCGCCCCTTGTCC	TCCTGGCTGGCCCGAGTGC
*Pparg*	CCTGCGGAAGCCCTTTGGTGAC	CTTGGCGAACAGCTGGGAGGAC
*Tbx3*	CTGCCCTTCCACCTCCAACAACAC	GGCACCGGGACTGGGATGGAATAC
*Albumin*	CAAGGCTGCCGACAAGGATAAC	AATTGCGGCACAGAGAAAAGAAGT
*Cyp3a11*	AGAGAACCGCATGCAAGAGAAAGT	AGCCGGCAAAAATAAAGAAAATGG
*G6pC*	TCGGGGGAATGCATGTGATAGGAA	CCCCGGATGTGGCTGAAAGTT
*Cd44*	GACCGGGATGACGCCTTCTTTATT	TTCTTGCCTCTTGGGTGGTGTTTC
*Prom1*	AGGGGGTATATGTCGATTGATGAA	ATTAAGCCACCCAGCCACCAGTAT
*Sox9*	TGGCAGAGGGTGGCAGACAGC	CGTTGGGCGGCAGGTATTGG
