# Supplementary material for: Generation and characterization of rat liver stem cell lines and their engraftment in a rat model of liver failure
Source: Sci Rep. 2016 Feb 26;6:22154. doi: 10.1038/srep22154 (PMC4768167; doi:10.1038/srep22154)
Supplement: Supplementary Information [file srep22154-s1.pdf]

Generation and characterization of rat liver stem cell lines and their engraftment in a rat model of liver failure

Ewart W. Kuijk[1*], Shauna Rasmussen[2*], Francis Blokzijl[1], Meritxell Huch[3], Helmuth Gehart[1], Pim Toonen[1], Harry Begthel[1], Hans Clevers[1], Aron M. Geurts[2,4], Edwin Cuppen[1]

Supplemental figures

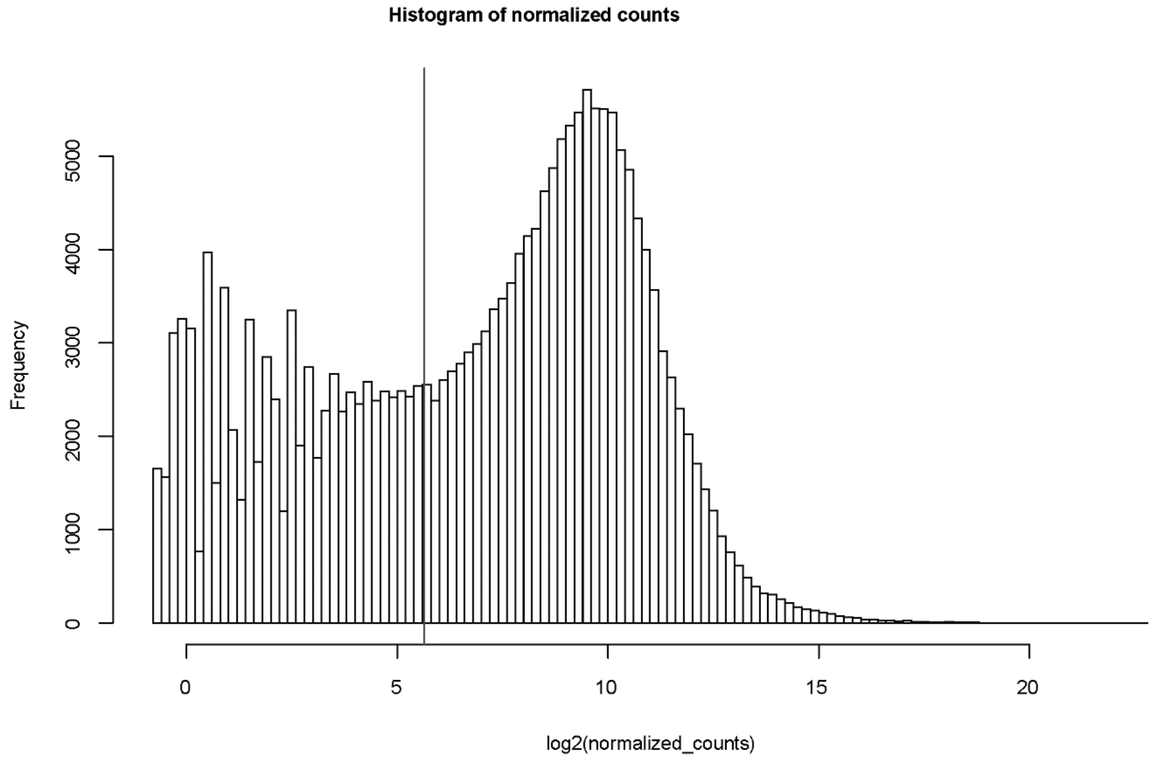

Supplemental figure S1: Histogram of normalized counts

Histogram of the non-zero normalized gene counts shows a bimodal distribution. Vertical line indicates threshold (50) of expression, which was used to select genes for Spearman’s rank correlation.

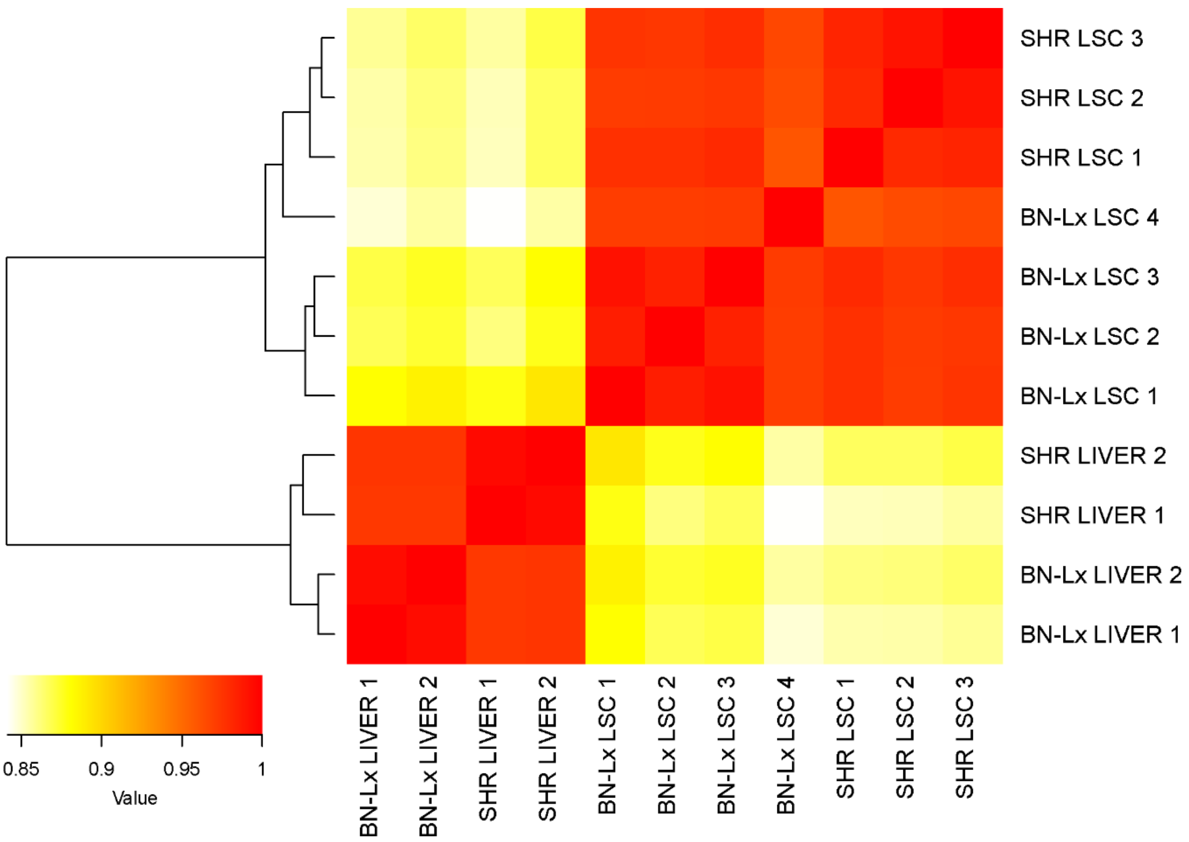

Supplementary figure S2: Hierarchical clustering of samples used for RNA-seq

Hierarchical clustering of samples used for RNA-seq based on the Spearman's rank correlation between the samples using the normalized counts of expressed genes. Color indicates Spearman's rank correlation between the samples, and hierarchical clustering divides liver and liver stem cell (LSC) samples into two groups.
